# Transport genes and chemotaxis in *Laribacter hongkongensis*: a genome-wide analysis

**DOI:** 10.1186/2045-3701-1-28

**Published:** 2011-08-17

**Authors:** Susanna KP Lau, Rachel YY Fan, Gilman KM Wong, Jade LL Teng, Kong-Hung Sze, Herman Tse, Kwok-Yung Yuen, Patrick CY Woo

**Affiliations:** 1 State Key Laboratory of Emerging Infectious Diseases, Hong Kong; 2 Research Centre of Infection and Immunology, The University of Hong Kong, Hong Kong; 3 Carol Yu Centre for Infection, The University of Hong Kong, Hong Kong; 4 Department of Microbiology, The University of Hong Kong, Hong Kong; 5 Department of Chemistry, The University of Hong Kong, Hong Kong

## Abstract

**Background:**

*Laribacter hongkongensis *is a Gram-negative, sea gull-shaped rod associated with community-acquired gastroenteritis. The bacterium has been found in diverse freshwater environments including fish, frogs and drinking water reservoirs. Using the complete genome sequence data of *L. hongkongensis*, we performed a comprehensive analysis of putative transport-related genes and genes related to chemotaxis, motility and quorum sensing, which may help the bacterium adapt to the changing environments and combat harmful substances.

**Results:**

A genome-wide analysis using Transport Classification Database TCDB, similarity and keyword searches revealed the presence of a large diversity of transporters (n = 457) and genes related to chemotaxis (n = 52) and flagellar biosynthesis (n = 40) in the *L. hongkongensis *genome. The transporters included those from all seven major transporter categories, which may allow the uptake of essential nutrients or ions, and extrusion of metabolic end products and hazardous substances. *L. hongkongensis *is unique among closely related members of *Neisseriaceae *family in possessing higher number of proteins related to transport of ammonium, urea and dicarboxylate, which may reflect the importance of nitrogen and dicarboxylate metabolism in this assacharolytic bacterium. Structural modeling of two C^_4_^-dicarboxylate transporters showed that they possessed similar structures to the determined structures of other DctP-TRAP transporters, with one having an unusual disulfide bond. Diverse mechanisms for iron transport, including hemin transporters for iron acquisition from host proteins, were also identified. In addition to the chemotaxis and flagella-related genes, the *L. hongkongensis *genome also contained two copies of *qseB/qseC *homologues of the AI-3 quorum sensing system.

**Conclusions:**

The large number of diverse transporters and genes involved in chemotaxis, motility and quorum sensing suggested that the bacterium may utilize a complex system to adapt to different environments. Structural modeling will provide useful insights on the transporters in *L. hongkongensis*.

## Background

*Laribacter hongkongensis *is a Gram-negative, sea gull-shaped, rod that belongs to the *Neisseriaceae* family of β-proteobacteria [1,2]. The bacterium was first isolated from the blood and empyema pus of a man with alcoholic cirrhosis and bacteremic empyema thoracis in Hong Kong [1]. Using the selective medium, cefoperazone MacConkey agar, the bacterium was subsequently isolated from the stool of patients with gastroenteritis [3,4]. In a multicenter case-control study, *L. hongkongensis *was shown to be associated with community-acquired gastroenteritis, with recent travel and eating fish being risk factors [5]. Apart from the human gut, *L. hongkongensis *has also been isolated from gut of freshwater animals including fish and Chinese tiger frogs as well as water from drinking water reservoirs [2,5-9]. In order to adapt to the changing environments and intestines of different animal hosts including human, fish and amphibians, *L. hongkongensis *must possess mechanisms to combat harmful substances in the environment and immune defense of animal hosts.

Transport-related proteins of bacteria are important in allowing the uptake of essential nutrients or ions, and extrusion of metabolic end products and hazardous substances. Bacteria employ different mechanisms for transport of different chemicals and these mechanisms have been classified into seven major categories according to the Transport Protein Database (TCDB): channels and pores (class 1), electrochemical potential-driven transporters (class 2), primary active transporters (class 3), group translocators (class 4), transmembrane electron carriers (class 5), accessory factors involved in transport (class 8), and incompletely characterized transport systems (class 9).

Bacteria also possess sophisticated signaling systems to sense and adapt to various substances in the environment. Depending on whether the environmental substances are attractants or repellents, the bacterium may migrate towards or away from the substances, which include certain amino acids, sugars, and metal ions [10-12]. This sense-and-swim ability is important for bacteria to be able to find the suitable environment for optimal growth. Chemotaxis involves two separate systems, the chemoreceptors located in the bacterial cell membrane which are important for sensing the binding compounds, and the transduction proteins which are involved in the downstream signal transduction in response to the stimuli. The chemoreceptors are also called methyl-accepting chemotaxis proteins (MCPs), which are reversibly methylated and function as homodimers [11,13].

The availability of the complete genome sequence of *L. hongkongensis *has allowed an opportunity to study its biology and important factors for adaptation to the changing environment [14]. We have previously found that transport-related proteins, including all seven major categories of transporters, account for about 14.1% of all coding sequences in the *L. hongkongensis *genome, suggesting that this group of proteins may be important for survival of the bacterium in the various environments and hosts [14]. Genes related to motility and chemotaxis were also identified [14]. Except for the first strain isolated from blood culture and empyema pus of a patient which was likely a non-motile variant, all strains from patients with gastroenteritis, animals or environmental water samples are motile with polar flagellae [1,4-7,10], suggesting that chemotaxis and motility may be an important mechanism for environmental adaptation in most isolates of *L. hongkongensis*. In this study, a comprehensive analysis of putative transport-related genes and genes related to chemotaxis, motility and quorum sensing in the *L. hongkongensis *genome is performed.

## Results and discussion

### Transport genes in L. hongkongensis genome

A huge diversity of transporters, including those from all seven major categories, were identified in the *L. hongkongensis *genome, as described in our previous complete genome report [14]. This may reflect its ability to adapt to various environments, including freshwater animals, water and human intestines. These transporters included: (1) 48 channels and pores, (2) 134 electrochemical potential-driven transporters, (3) 194 primary active transporters, (4) 9 group translocators, (5) 16 transmembrane electron carriers, (6) 7 accessory factors involved in transport and (7) 49 transporters of incompletely characterized transport systems (Table 1).

**Table 1:** Transporters in *L. hongkongensis *and *C. violaceum*

Category	*L. hongkongensis*			*C. violaceum*		
	**No. of CDSs**	**% of total CDSs**	**% of transport CDSs**	**No. of CDSs**	**% of total CDSs**	**% of transport CDSs**
Channel and Pores	48	1.5	10.5	63	1.4	11.3
α-type channels	17			26		
β-barrel porins	29			43		
Pore-forming toxins (proteins and peptides)	0			3		
Holins	2			2		
Electrochemical Potential-driven Transporters	134	4.1	29.3	161	3.7	28.8
Porters (uniporters, symporters and antiporters)	132			159		
Ion-gradient-driven energizers	2			2		
Primary Active Transporters	194	6.0	42.5	252	5.7	45.0
P-P-bond-hydrolysis-driven transporters	150			206		
Decarboxylation-driven transporters	5			7		
Oxidoreduction-driven transporters	39			39		
Group Translocators	9	0.3	2.0	18	0.4	3.2
Phosphotransfer-driven group translocators	2			8		
Acyl CoA ligase-coupled transporters	7			10		
Transmembrane Electron Carriers	16	0.5	3.5	13	0.3	2.3
Transmembrane 2-electron transfer carriers	14			12		
Transmembrane 1-electron transfer carriers	2			1		
Accessory Factors Involved in Transport	7	0.2	1.5	20	0.5	3.6
Auxiliary transport proteins	7			20		
Incompletely Characterized Transport Systems	49	1.5	10.7	33	0.7	5.9
Recognized transporters of unknown biochemical mechanism	15			14		
Putative transport proteins	34			19		

#### Channels and pores

The outer membranes of lipid bilayer envelopes of Gram-negative bacteria contain large numbers of water-filled transmembrane protein channels known as porins [15]. They serve as a molecular filter allowing for permeation of hydrophilic molecules up to a certain size or specific solutes into the periplasmic space. Some bacterial porins also serve as receptor for phage and bacteriocin binding [16]. X-ray crystoallography studies and atomic structures have revealed that porin molecules exists as trimers, with the transmembrane core composed of mostly β-sheets and some α-helixes [15]. The *L. hongkongensis *genome contained 48 coding sequences (CDSs) belonging to channels and pores, of which 17 were α-type channels, 29 were β-barrel porins and 2 were holins (Table 1).

Among the 17 α-type channels, five were mechanosensitive channels, including one large conductance mechanosensitive channel (LHK_02562) and four small conductance mechanosensitive channels (LHK_01830, LHK_01942, LHK_02394 and LHK_02965), which are responsible for mediating resistance to mechanophysical changes [17]. Interestingly, three CDSs encoding proteins of the ammonium transporter family were identified in the *L. hongkongensis *genome, as compared to only one copy such genes in *Chromobacterium violaceum*, the most closely related bacterial species of the *Neisseriaceae *family with complete genome sequence available (Table 2). Moreover, a homologue of urea transporter responsible for urea uptake (LHK_01044) was also present in *L. hongkongensis *(Table 2), while this protein was absent in *C. violaceum *and the pathogenic *Neisseria *spp., *Neisseria gonorrhoeae *and *Neisseria meningitidis*. This may reflect the importance of nitrogen metabolism of the bacterium, as *L. hongkongensis *is assacharolytic and has been shown to use different pathways for arginine synthesis regulated at different temperatures [14]. In fact, the habitats of the closely related bacterial species are quite different from that of *L. hongkongensis*, where the latter can survive in human intestine in addition to diverse freshwater environment. This may also explain its unique ability in maximizing nitrogen metabolism. Among the β-barrel porins, the OmpA-OmpF-type porins are most well known in bacteria to allow passive diffusion of hydrophilic substrates across the outer membrane. Three CDSs coding for putative OmpA-OmpF-type porins were identified in the *L. hongkongensis *genome. Interestingly, two homologues of another β-barrel porin, fatty acid transporter gene (*fadL*), were also found, which may be important for uptake of long-chain fatty acids in freshwater environments poor in lipids or fatty acids.

**Table 2:** α-type channels in *L. hongkongensis *and their closest homologues

CDS	Protein	Closest match organism	Best E-value	Amino acid identity (%)
LHK_02933	Ammonium transporter	*L. nitroferrum*	2.00E-146	73.18
LHK_03249	Ammonium transporter	*Shewanella halifaxensis*	2.00E-118	62.32
LHK_03154	Ammonium transporter family protein	*L. nitroferrum*	1.00E-163	78.99
LHK_02207	Flagellar motor protein MotA	*L. nitroferrum*	1.00E-122	74.48
LHK_00970	Ion transporter	*C. violaceum*	5.00E-78	58.96
LHK_02562	Large-conductance mechanosensitive channel	*Pelodictyon luteolum*	2.00E-43	56.95
LHK_01830	Transmembrane protein	*C. violaceum*	2.00E-109	57.52
LHK_01942	Mechanosensitive ion channel protein	*Janthinobacterium *sp. Marseille	5.00E-79	41.26
LHK_02394	MscS Mechanosensitive ion channel	*L. nitroferrum*	7.00E-55	48.95
LHK_02965	Transporter, small conductance mechanosensitive ion channel family	*E. coli *O157:H7	5.00E-73	61.04
LHK_02739	Molecular chaperone DnaK	*C. violaceum*	0	85.98
LHK_02206	OmpA/MotB domain protein	*L. nitroferrum*	4.00E-97	75.46
LHK_01044	Urea transporter	*Methylobacterium extorquens *PA1	1.00E-65	50.46
LHK_00053	TolQ-related transport transmembrane protein	*C. violaceum*	1.00E-86	74.66
LHK_03174	TolR protein	*C. violaceum*	5.00E-30	51.88
LHK_00499	Probable exbB-like biopolymer transport	*C. violaceum*	4.00E-55	59.31
LHK_00498	Biopolymer transport *exbD *transmembrane protein	*Burkholderia pseudomallei*112	7.00E-36	55.88

#### Electrochemical potential-driven transporters

The *L. hongkongensis *genome possessed a large number of CDSs (n = 134) encoding for putative electrochemical potential-driven transporters, among which the majority (132 CDSs) were porters including uniporters, symporters and antiporters, while the remaining two CDSs were ion-gradient-driven energizers (Table 1). Of the 132 porters, 19 (14.3%) belonged to the major facilitator superfamily (MFS). MFS proteins are important transporters in bacteria, which allow transport of molecules by an electrochemical ion gradient and typically contain a single subunit with 12 membrane-spanning helixes [18]. The MFS proteins of *L. hongkongensis *were predicted to mediate transport of diverse substrates including ions, drugs and metabolites. Another major family of porters were the resistance-nodulation-cell division (RND) superfamily (28 CDSs), which are responsible for transporting a wide variety of substrates including antibiotics, dyes, detergents, fatty acids, bile salts, organic solvents, heavy metals, autoinducers and lipooligosaccharides in Gram-negative bacteria [19,20]. Other porters belonged to diverse families of proteins which facilitate the transport of diverse substances including ions, amino acids, drugs, heavy metal such as nickel and cobalt, nucleobase, C_4_-dicarboxylates and other metabolites. The presence of various porters may be involved in acquisition of essential substances for metabolism and bacterial resistance to environmental toxic substances including heavy metals. Interestingly, a total of 11 porters for dicarboxylate transport were found in *L. hongkongensis *genome, as compared to only 6 in *C. violaceum *and 1 each in *N. meningitidis *and *N. gonorrhoeae *genomes (Table 3). C_4_-dicarboxylates are intermediates in TCA cycle that can be utilized by bacteria as nonfermentable carbon and/or energy sources under aerobic or anaerobic conditions [21]. Some C_4_-dicarboxylates, such as succinate, oxalate and malate, can also be found in nature [22]. The presence of high number of C_4_-dicarboxylates transporters may reflect the ability of using C_4_-dicarboxylates as carbon sources in *L. hongkongensis*, as the bacterium is assacharolytic, lacking a complete glycolytic pathway, and is in line with our experiments showing that L-malate can be used as its sole carbon source [14].

**Table 3:** Porters for dicarboxylates in *L. hongkongensis *and related bacteria

Family	*L. hongkongensis*	*C. violaceum*	*N. meningitidis*	*N. gonorrhoeae*
C4-Dicarboxylate Uptake (Dcu) Family	0	2	0	0
Dicarboxylate/Amino Acid:Cation (Na or H) Symporter (DAACS) Family	3	1	0	0
Tripartite ATP-independent Periplasmic Transporter (TRAP-T) Family	6	3	0	0
Divalent Anion:Na+ Symporter (DASS) Family	1	0	1	1
C_4_-dicarboxylate Uptake C (DcuC) Family	1	0	0	0
Total	11	6	1	1

**Figure 1:**
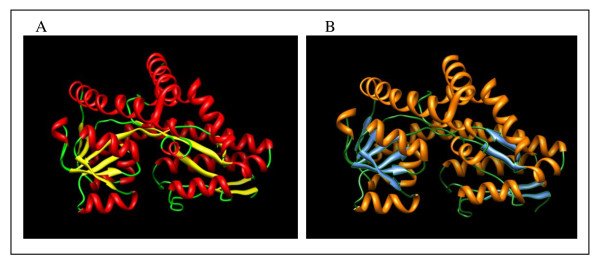
**Homology model of DctP_00983 (panel A) and DctP_01394 (panel B), putative DctP TRAP transporters for C_4_-dicarboxylate in *L. hongkongensis***. For DctP_00983, the C-score of the model was 1.49, which approximately corresponded to an expected TM-score of 0.92 ± 0.06 and an expected root-mean-square deviation (RMSD) of 3.2 ± 2.3 Å from the native structure. The Ramachandran plot showed that 99.6% of aa are in the favored and allowed regions. Calculated G-factors for dihedral angles and main-chain covalent forces are 0.11 and -0.17 respectively, with an overall average of 0.01. The Z-score of the model is -7.84, which is comparable to other experimentally determined protein chains of a similar size in the PDB. Local model quality analysis by plot of residue scores in ProSA-web did not reveal any problematic regions in the structure. The quality analysis results suggested that the homology model is mostly reliable with good structural qualities. For DctP_01394, the C-score of the model was 1.36, which approximately corresponded to an expected TM-score of 0.90 ± 0.06 and an expected RMSD of 3.5 ± 2.4 Å from the native structure. The Ramachandran plot showed that 99.0% of aa are in the favored and allowed regions. Calculated G-factors for dihedral angles and main-chain covalent forces are 0.09 and -0.17 respectively, with an overall average of 0.00. The Z-score of the model is -8.15, which is comparable to other experimentally determined protein chains of a similar size in the PDB. Local model quality analysis by plot of residue scores in ProSA-web did not reveal any problematic regions in the structure. The quality analysis results suggested that the homology model of DctP_01394 is also reliable with good structural qualities.

**Figure 2:**
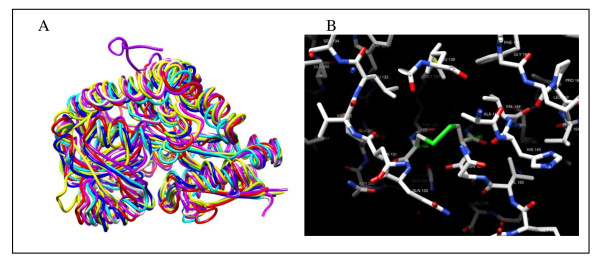
**Structural alignment of the homology model of DctP_00983 and DctP_01394, showing similar structures to other DctP-type SBPs (panel A) and a disulfide bond predicted between the cysteine residues at positions 129 and 182 of DctP_00983 (panel B)**. RMSD between DctP_00983 and the related structures ranged from 0.761 to 1.290 Å. RMSD between DctP_01394 and the related structures ranged from 0.891 to 1.377 Å.

Six of the 11 porters for dicarboxylate transport found in *L. hongkongensis *genome were believed to form two DctP-type tripartite ATP-independent periplasmic (TRAP) transporters which belong a heterogeneous group of substrate-binding protein (SBP)-dependent secondary transporters of a diverse range of substrates found in bacteria and archaea [23-25]. The genes encoding the 3 subunits were arranged in an operon, with two membrane proteins DctQ and DctM associating with DctP to form a C_4_-dicarboxylate TRAP transporter [26]. Several TRAP transporters have been characterized in detail, with the structures of at least seven DctP-type SBP subunits determined [25]. These studies revealed significant structural and architectural similarities among the different SBPs, while highlighting the differences that permitted these proteins to bind their respective substrates with high affinity and specificity. Besides substrate recognition, it was also found that the SBP performs other essential functions [27], and likely interacts with the integral membrane components in a hitherto undiscovered manner. One operon (LHK_00983-00984-00985), encoding C_4_-dicarboxylate transporter, was found downstream of several genes related to allantoin regulation and utilization; while the other operon (LHK_01394-01393-01392) was located upstream of the *maeB *gene encoding NADP-dependent malate dehydrogenase. The SBP encoded by LHK_00983 (DctP_00983) was a 331 aa protein containing a 22 aa N-terminal signal peptide, with a predicted molecular weight of 33.9 kDa. It possessed 48% amino acid identity to the closest homolog in *Roseovarius *sp. TM1035 (NCBI accession no.: ZP_01881277). The SBP encoded by LHK_01394 (DctP_01394) was a 335 aa protein containing a 24 aa N-terminal signal peptide, with a predicted molecular weight of 34.3 kDa. It possessed 74% amino acid identity to the closest homolog in *C. violaceum *ATCC12472. The homology model and structural alignment of the homology model showed that the overall structure of DctP_00983 and DctP_01394 was very similar to the determined structures of other DctP-type SBPs (Figure 1 and 2, and see Supplementary material). Similar to other DctP homologs, they were divided into two domains with conserved arrangements of α-helices and β-sheets, which are connected by a characteristic hinge made up of two β-strands and an α-helix. A highly conserved arginine residue in domain II is present in both proteins (Arg145 of DctP_00983 and Arg147 of DctP_01394), which corresponds to Arg147 in SiaP of *H. influenzae *essential to SBP function by forming a salt bridge with the carboxylate group of the ligand [28]. Interestingly, a disulfide bond was predicted between the cysteine residues at positions 129 and 182 for DctP_00983 (Figure 2) by homology modeling and sequence analysis. This structural feature was also found in the closest homolog in *Roseovarius *sp. TM1035, but absent from other related DctP-type SBP homologs including DctP_01394.

#### Primary active transporters

Primary active transporters mediate energy-driven transport of substances in and out of bacterial cells by using ATP hydrolysis, photon absorption, electron flow, substrate decarboxylation, or methyl transfer [29]. Primary active transporters were the most abundant class of transporters (194 CDSs), constituting 6% of CDSs in the *L. hongkongensis *genome, among which 150 belonged to P-P-bond-hydrolysis-driven transporters (Table 1). Of the 150 P-P-bond-hydrolysis-driven transporters, 109 were ATP-binding cassette (ABC) transporters which are one of the largest groups of membrane proteins using energy from ATP hydrolysis for transport. In bacteria, they reside in the inner membrane and are involved in both uptake and export of a wide range of substances. All ABC transporters share a common basic structure which consists of four domains: two transmembrane domains, typically with six transmembrane spans per domain, and two cytoplasmic nucleotide-binding domains which catalyse nucleotide hydrolysis [30]. In bacteria, these domains are encoded as separate polypeptides. Determined by the structure of the transmembrane domain, ABC transporters are typically specific for the substrates that they are responsible for, although some may transport for multiple related substances. As a result, the numbers of ABC transporters in different bacterial species vary widely, depending on its need for adaptation to varying environmental conditions [31]. The ABC transporters in the *L. hongkongensis *are likely involved in the active transport of diverse substances, including carbohydrate, amino acids or peptides, ions, vitamins, lipids, drugs and heavy metals including molybdenum, iron, zinc, cobalt, magnesium, copper, cadmium, mercury, lead, arsenite and nickel. These systems were often arranged in gene clusters comprising the ATP-binding protein and two auxiliary proteins, a permease and a substrate-binding protein. Compared to the 70 ABC transporters found in *E. coli *[31], the *L. hongkongensis *genome contained a large number of such proteins, reflecting its ability to adapt to different hosts and environment.

Apart from P-P-bond-hydrolysis-driven transporters, other primary active transporters identified in the *L. hongkongensis *genome included oxidoreduction-driven transporters (39 CDSs) and decarboxylation-driven transporters (5 CDSs), which use chemical energy to perform transport of charged or uncharged molecules across the membrane against the concentration gradient [32].

#### Group translocators

Of the nine group translocators, two were phosphotransfer-driven group translocators and seven were acyl CoA ligase-coupled transporters belonging to the fatty acid transporter (FAT) family. The phosphotransferase group translocators are components of the bacterial phosphotransferase system (PTS), which catalyzes translocation of sugars and hexitols with concomitant phosporylation, and regulates the metabolism in response to the availability of carbohydrates. PTSs consist of two cytoplasmic proteins, enzyme I (EI) and HPr, and a variable number of sugar-specific transport complexes (Enzymes II^sugar^) belonging to the group translocators. While the *Escherichia coli *genome encoded 38 different PTS proteins, the *L. hongkongensis *genome encoded only one gene for EI and HPr each and two genes for transporters, one containing protein-N p-phosphohistidine-sugar phosphotransferase IIA domain and the other containing nitrogen-regulatory fructose-specific IIA domain [33]. This is likely related to the relative unimportance of sugar metabolism in *L. hongkongensis*.

#### Transmembrane electron carriers

There were 16 transmembrane electron carriers in the *L. hongkongensis *genome, including 14 transmembrane 2- and two transmembrane 1-electron transfer carriers. Among the 14 transmembrane 2-electron transfer carriers, 12 belonged to the prokaryotic molybdopterin-containing oxidoreductase (PMO) family, and the other 2 belonged to the disulfide bond oxidoreductase D (DsbD) and B (DsbB) family respectively.

#### Accessory factors involved in transport

There were seven accessory factors belonging to auxiliary transport proteins in the *L. hongkongensis *genome, 3 belonging to the membrane fusion protein (MFP) family, 2 to the phosphotransferase system enzyme I (EI) family, 1 to the phosphotransferase system HPr (HPr) family and 1 to the stomatin/podocin/band 7/nephrosis.2/SPFH (stomatin) family.

#### Incompletely characterized transport systems

Of the 49 CDSs belonging to incompletely characterized transport system, 15 were recognized transporters of unknown biochemical mechanism, with 6 belonging to the putative type VI symbiosis/virulence secretory pathway (VISP) family, 2 to the HlyC/CorC (HCC) family, 2 to the capsular polysaccharide exporter (CPS-E) family, 1 to the tellurium ion resistance (TerC) family and the remaining 4 being metal ion transporters. The other 34 CDSs were putative transport proteins, including 2 CDSs of the camphor resistance (CrcB) family and 1 probable hemolysin III.

### Iron Transport in L. hongkongensis

Iron is an essential metal for most microorganisms used in many key molecules involved in metabolism. In bacteria, iron metabolism has been shown to be important in adaptation to the environment especially within the host and as a result related to virulence. Diverse mechanisms for iron transport were identified in the *L. hongkongensis *genome, suggesting that the bacterium is able to adapt to iron limitation present in human body which represents one of the non-specific immune response called induced hypoferremia [34,35]

#### Siderophores and iron uptake

Siderophores are low molecular mass compounds with high affinity for ferric iron. In contrast to *C. violaceum *which produced siderophores for iron acquisition, proteins related to siderophore production were not found in *L. hongkongensis *genome. However, a homolog of TonB-dependent siderophore receptor (LHK_00497) was present, as described in our previous report [14]. Although *Listeria monocytogenes *also did not produce siderophores for iron acquisition, it was able to obtain iron by using either exogenous siderophores produced by various microorganisms or natural catechol compounds widespread in the environment [36,37]. It remains to be determined if *L. hongkongensis *can utilize exogenous siderophores or other natural iron-binding compounds for iron acquisition.

#### Hemin transport

Despite the inability to produce siderophores, a set of genes related to the transport of hemin were identified in *L. hongkongensis *genome (8 CDSs compared to 6 CDSs in *C. violaceum*). The 8 CDSs included TonB-dependent receptor (LHK_01193), hemin degrading factor (LHK_01192), ABC transporter permease (LHK_01189), ferric citrate transport system ATP-binding protein (LHK_01188), hemin-binding periplasmic protein (LHK_01190), hemin importer ATP-binding subunit (LHK_01427), hemin ABC transporter permease protein (LHK_01428) and Fur family ferric uptake regulator (LHK_01431). The conserved domains for hemin receptor, FRAP and NPNL, were also identified in the TonB-dependent receptor [38]. This suggests that *L. hongkongensis *is able to utilize iron source form host proteins, which may be important for survival in its hosts. Three other CDSs, homologous to *fbpA *(LHK_02634), *fbpB *(LHK_02635) and ATP-binding protein (LHK_02636), ABC transporters for transferrin and lactoferrin, were also present, although the outer membrane receptor is not found.

#### ABC transporters of the metal type

A cluster of three genes encoding an ABC transporter of the metal type (homologous to that identified in *C. violaceum*) was identified in the *L. hongkongensis *genome. They encoded a periplasmic Mn^2+^/Zn^2+^-binding (lipo)protein (surface adhesion A) (*znuA*), a Mn^2+^/Zn^2+ ^permease component (*znuB*) and the ATPase component (*znuC*). In addition, a gene encoding a putative cadmium-translocating ATPase component (cadmium-translocating P-type ATPase) (CadA) (LHK_00449) was also present. A similar gene was also found in *C. violaceum *(CV1154), which was thought to be a surface adhesion A component for Mn^2+^/Zn^2+ ^binding. The Fur family ferric uptake regulator (*zur*) (LHK_01344) was also present.

#### Other transporters

In addition to the above transporters, two CDSs encoding ferrous iron transport proteins, *feoA *(LHK_03044) and *feoB *(LHK_03045), were identified in *L. hongkongensis *genome, which are believed to provide iron supply under anaerobic or low pH conditions in bacteria [39]. Three other CDSs homologous to iron uptake ABC transporter periplasmic solute-binding protein (LHK_01590), ABC transporter permease (LHK_01593) and ABC transporter ATP-binding protein (LHK_01591) were also found.

#### Iron storage

Mechanism required for storage of iron after its acquisition from the environment was present in *L. hongkongensis*, which mainly depends on two proteins: bacterioferritin (BFR) (LHK_01239, homologous to CV3399 in *C. violaceum*) and frataxin-like homolog (LHK_00023, homologous to Daro_0208 in *Dechloromonas aromatica*). The BFR is an iron-storage protein with close similarity to the ferritins found in both eukaryotes and prokaryotes [40]. The frataxin-like homolog has been implicated in iron storage in other bacteria. The frataxin-like domain is related to frataxin, the protein mutated in Friedreich's ataxia which is therefore proposed to result from decreased mitochondrial iron storage [41,42].

#### Regulation of iron transport

Fur protein is a global repressor protein by forming Fur-Fe^2+ ^complexes that bind to iron-dependent promoter during iron-rich conditions. It regulates ferrichrome (*fhuABCDG*), ferric citrate (*fecABCDE*) and ferrous iron (*feoABC*) uptake systems. The Fur protein in *L. hongkongensis *was encoded in CDS LHK_01431 (homologous to FuraDRAFT_2340 in *Lutiella nitroferrum*).

### Chemotaxis in L. hongkongensis

#### Methyl-accepting chemotaxis and chemosensory transducer proteins

A total of 52 open reading frames (CDSs) were related to chemotaxis, of which 29 encoded MCPs and 22 were chemosensory transducer proteins. Most genes encoding MCPs were scattered throughout the *L. hongkongensis *genome, while the genes encoding transducer proteins were mostly arranged in three gene clusters as described in our previous report (Table 4) [14].

**Table 4:** CDSs related to chemotaxis in *L. hongkongensis *genome

CDS	Gene	Product	Organism with the closest matching sequences	E-value	Identities	Cluster*^a^*
LHK_00115		histidine kinase, HAMP region: chemotaxis sensory transducer	*D. aromatica*	1e-96	242/680 (35%)	
LHK_00482		methyl-accepting chemotaxis sensory transducer	*L. nitroferrum*	4e-55	164/543(30%)	
LHK_00516		methyl-accepting chemotaxis sensory transducer	*L. nitroferrum*	8e-129	265/513 (51%)	
LHK_00553		diguanylate phosphodiesterase	*C. violaceum*	6e-111	211/406 (51%)	CA
LHK_00554	*cheA1*	CheA signal transduction histidine kinase	*L. nitroferrum*	0	443/613 (72%)	CA
LHK_00555	*cheZ1*	chemotaxis phosphatase, CheZ	*L. nitroferrum*	2e-69	139/244(59%)	CA
LHK_00556	*cheY1*	chemotaxis regulator protein CheY	*C. violaceum*	4e-61	109/130 (83%)	CA
LHK_00557	*cheV1*	chemotaxis protein CheV	*C. violaceum*	1e-138	240/314 (76%)	CA
LHK_00558	*cheV2*	chemotaxis protein CheV	*C. violaceum*	5e-147	251/313 (80%)	CA
LHK_00559		two-component sensor histidine kinase	*L. nitroferrum*	2e-59	169/381 (44%)	CA
LHK_00560		chemotaxis sensory transducer	*D. aromatica*	6e-24	100/320 (31%)	CA
LHK_00561	*cheY2*	chemotaxis protein cheY	*D. aromatica*	8e-46	85/121 (70%)	CA
LHK_00562	*cheA2*	chemotaxis protein CheA	*C. violaceum*	2e-161	358/746 (47%)	CA
LHK_00563	*cheW*	CheW protein	*Burkholderia phytofirmans*	1e-40	95/153 (62%)	CA
LHK_00564		methyl-accepting chemotaxis protein	*C. violaceum*	4e-143	315/475 (66%)	CA
LHK_00565	*cheR*	CheR chemotaxis protein methyltransferase	*Janthinobacterium *sp. Marseille	5e-68	125/273 (45%)	CA
LHK_00566	*cheB1*	chemotaxis-specific methylesterase	*Nitrosomonas europaea*	2e-99	186/355 (52%)	CA
LHK_00567	*cheD*	chemoreceptor glutamine deamidase CheD	*D. aromatica*	5e-59	108/189 (57%)	CA
LHK_00603		methyl-accepting chemotaxis protein	*C. violaceum*	7e-103	242/624 (38%)	
LHK_00617		methyl-accepting chemotaxis protein IV	*C. violaceum*	2e-100	223/481 (46%)	
LHK_00700		methyl-accepting chemotaxis sensory transducer	*Allochromatium vinosum*	0	384/715 (53%)	
LHK_00726	*aer1*	methyl-accepting chemotaxis sensory transducer with Pas/Pac sensor	*L. nitroferrum*	7e-114	232/528 (43%)	
LHK_00935	*cheR*	MCP methyltransferase, CheR-type	*L. nitroferrum*	2e-92	170/282 (60%)	
LHK_01020		putative aromatic hydrocarbon chemotaxis transducer	*Azoarcus *sp.	4e-62	140/338 (41%)	
LHK_01116		methyl-accepting chemotaxis protein	*Denitrovibrio acetiphilus*	1e-59	152/461 (32%)	
LHK_01212		methyl-accepting chemotaxis sensory transducer	*L. nitroferrum*	1e-135	261/476 (54%)	
LHK_01359	*cheY3*	chemotaxis regulator protein CheY	*C. violaceum*	1e-56	102/127 (80%)	CB
LHK_01360	*cheV3*	chemotaxis protein CheV	*C. violaceum*	1e-134	231/309 (74%)	CB
LHK_01361		methyl-accepting chemotaxis sensory transducer	*L. nitroferrum*	6e-47	157/506 (31%)	CB
LHK_01372		chemotaxis sensory transducer	*D. aromatica*	4e-49	166/534 (31%)	
LHK_01470		putative aromatic hydrocarbon chemotaxis transducer	*Azoarcus *sp.	2e-93	222/539 (41%)	
LHK_01602		methyl-accepting chemotaxis sensory transducer	*L. nitroferrum*	0	339/601 (56%)	
LHK_01618		methyl-accepting chemotaxis sensory transducer	*L. nitroferrum*	2e-87	209/525 (39%)	
LHK_01706		methyl-accepting chemotaxis protein IV	*C. violaceum*	1e-121	247/481 (51%)	
LHK_01721		methyl-accepting chemotaxis protein	*C. violaceum*	4e-113	240/627 (38%)	
LHK_02037		methyl-accepting chemotaxis sensory transducer	*Leptospirillum ferrodiazotrophum*	5e-63	137/327 (41%)	
LHK_02158	*aer2*	methyl-accepting chemotaxis sensory transducer with Pas/Pac sensor	*Ralstonia pickettii*	6e-39	98/276 (35%)	
LHK_02165		methyl-accepting chemotaxis protein	*C. violaceum*	8e-146	275/631 (43%)	
LHK_02364	*cheB2*	response regulator receiver modulated CheB methylesterase	*Geobacter bemidjiensis*	1e-63	122/206 (59%)	
LHK_02427		methyl-accepting chemotaxis protein	*C. violaceum*	6e-110	227/629 (36%)	CC
LHK_02428		Hypothetical protein	No			CC
LHK_02429	*cheV4*	response regulator receiver modulated CheW protein	*L. nitroferrum*	2e-145	248/313 (79%)	CC
LHK_02430	*cheV5*	chemotaxis protein CheV	*C. violaceum*	3e-137	237/314 (75%)	CC
LHK_02431	*cheY4*	chemotaxis regulator protein CheY	*C. violaceum*	2e-58	105/127 (82%)	CC
LHK_02432	*cheZ2*	chemotaxis phosphatase, CheZ	*L. nitroferrum*	2e-63	129/248 (52%)	CC
LHK_02433	*cheA3*	CheA signal transduction histidine kinase	*L. nitroferrum*	0	420/611 (68%)	CC
LHK_02455		methyl-accepting chemotaxis sensory transducer	*Candidatus Accumulibacter phosphates*	1e-74	154/326 (47%)	
LHK_02575		putative Methyl-accepting or sensory transducer chemotaxis protein	*Alteromonadales bacterium*	1e-83	172/407 (42%)	
LHK_02814	*aer3*	chemotaxis sensory transducer	*Rhodopseudomonas palustris*	8e-42	138/425 (32%)	
LHK_02834		methyl-accepting chemotaxis protein	*Pseudomonas syringae*	1e-45	148/437 (33%)	
LHK_03026		methyl-accepting chemotaxis protein	*C. violaceum*	6e-145	275/627 (43%)	
LHK_03119		methyl-accepting chemotaxis sensory transducer	*L. nitroferrum*	2e-133	273/514 (53%)	
LHK_03163		methyl-accepting chemotaxis sensory transducer	*Candidatus Accumulibacter phosphatis*	9e-50	167/494 (33%)	

*^a^*The Che proteins were encoded in three gene clusters, named CA, CB and CC (chemotaxis A, B and C clusters)

All the predicted MCPs in *L. hongkongensis *possessed a transmembrane domain, which is compatible with their anticipated location in the bacterial cell membrane and function as receptors. Conserved domain structures were also identified in some of the MCPs. The plasmid achromobacter secretion (PAS) domain was found in four MCPs (LHK_00564, LHK_00726, LHK_02158 and LHK_02814). PAS domains are energy-sensing modules that are found in proteins from archaea to humans [43]. The histidine kinase adenylyl cyclase MCP and phosphatase (HAMP) domain was present in 22 of the 29 MCPs. The HAMP domain interacts with the PAS domain for signal transduction in aerotaxis (oxygen-sensing) receptor in *Escherichia coli *[43], and possesses roles of regulating the phosphorylation or methylation of homodimeric receptors by transmitting the conformational changes in periplasmic ligand-binding domains to cytoplasmic signaling kinase and methyl-acceptor domains [44].

**Figure 3:**
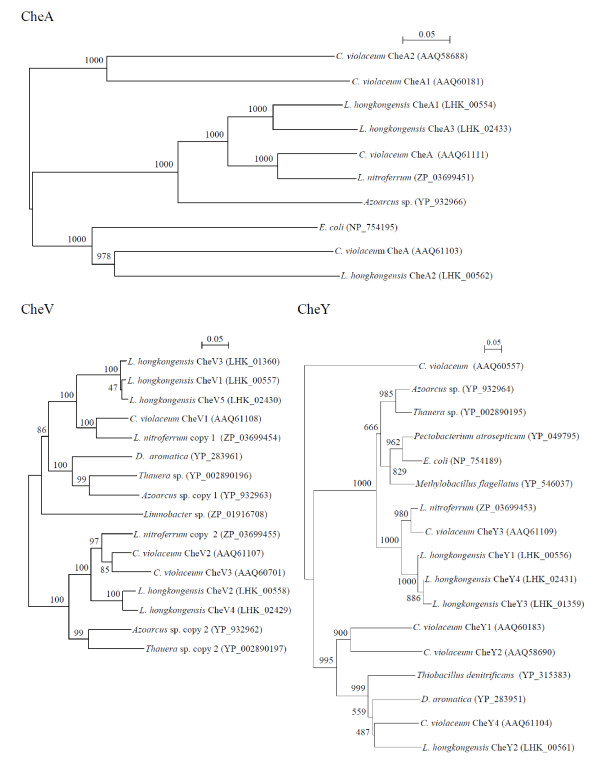
**Phylogenetic tree showing the relationships of the CheAs, CheVs and CheYs from *L. hongkongensis *to those from other bacteria**. The unrooted trees are constructed by using the neighbor-joining method using Kimura's two-parameter correction, with bootstrap values calculated from 1000 trees. The scale bar indicates the estimated number of substitutions per 20 bases. Bacterial names and accession numbers are given as cited in the GenBank database.

These chemosensory transducer proteins work as two-component regulatory systems which typically consist of a sensory histidine kinase and a response regulator. The histidine kinase is usually a transmembrane receptor and the response regulator a cytoplasmic protein [45]. Following autophosphorylation at a conserved histidine residue in response to changes in chemoreceptor occupancy, the histidine kinase serves as a phospho-donor for the response regulator. Once phosphorylated, the response regulator mediates changes in gene expression or cell motility. CheA is a typical sensory histidine kinase while CheY is a downstream regulator protein [46]. Upon phosphorylation, CheY binds to the FliM component at the base of the flagellar motor switch to induce clockwise rotation [47]. In contrast to the single copies of CheA and CheY in *E. coli*, the presence of 22 chemosensory transducer proteins, many with multiple copies including three CheA, one CheB, one CheD, two CheR, five CheV, one CheW, four CheY, and two CheZ, suggested that *L. hongkongensis *may utilize a complex transducer system to mediate chemotaxis response and adapt to environmental changes (Table 4). These Che proteins were encoded in three gene clusters, named CA, CB and CC. The first and largest cluster, CA, encoded two CheA, one CheR, two CheY, two CheV, one CheZ, and the single CheD and CheW. The second and smallest cluster, CB, encoded one CheV and CheY. The third cluster, CC, encoded one CheA, one CheY, two CheV and one CheZ. Phylogenetic analysis of CheAs, CheVs and CheYs of *L. hongkongensis *suggested that the multiple copies are the result of both horizontal transfer events and gene duplication, as some of the copies were more closely related to the corresponding proteins in other bacteria while others were more closely related among the homologues of *L. hongkongensis *(Figure 3).

**Figure 4:**
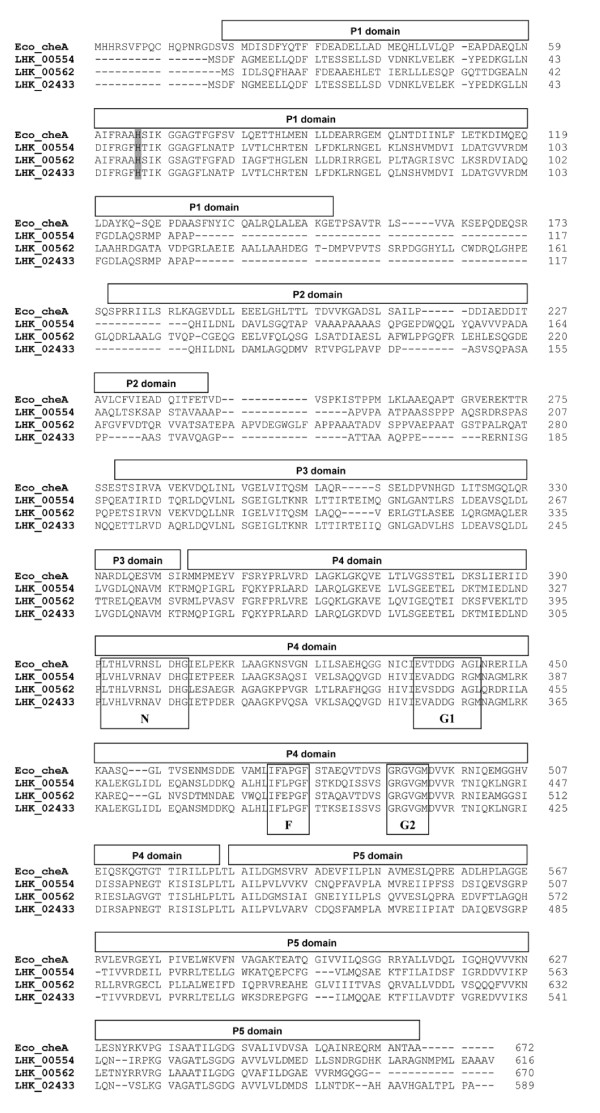
**Amino acid sequence alignments of *L. hongkongensis *and *E. coli *CheAs**. The conserved P1 to P5 domains are marked above the sequences. The histidine residue at potential phosphorylation site is shaded. The four conserved regions designated the N, G1, F and G2 boxes within P4 domain are marked in open boxes.

The CheA proteins of *L. hongkongensis *were most closely related to homologues in the closely related *Chromobacterium violaceum *and *Lutiella nitroferrum *with 47% to 72% amino acid identities. CheA has five domains, P1 to P5 [46]. All the three CheA proteins in *L. hongkongensis *contained these conserved domains. In the P1 domain, the invariant histidine residue, which undergoes phosphorylation by the P4 domain, was also present. In the kinase domain P4, the four conserved regions designated the N, G1, F and G2 boxes were also present in the three CheAs (Figure 4).

**Figure 5:**
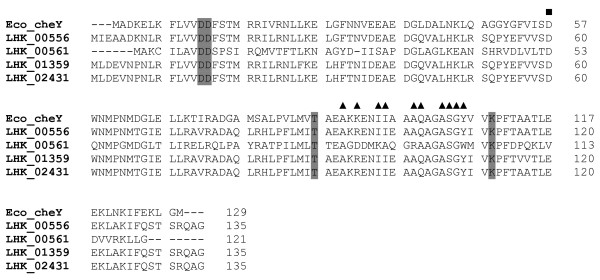
**Amino acid sequence alignments of *L. hongkongensis *and *E. coli *CheYs**. The conserved aspartate, threonine and lysine residues are shaded. The aspirate residue at potential phosphorylation site is marked by black square, and residues of *E. coli *CheY that interact with the P2 domain of *E. coli *Che A are marked by black triangles above the residues.

The CheY proteins of *L. hongkongensis *were highly similar to the homologues in *C. violaceum *and *Dechloromonas aromatica*, with 70% to 83% amino acid identities. Multiple alignment of the four CheY with that of *E. coli *showed the presence of all five amino acid residues conserved among response regulators [46,48]: aspartate at positions 12, 13 and 57; threonine at position 87, and lysine at position 109, with the aspartate at position 57 representing the phosphorylation site (Figure 5). Residues that interact with P2 domain of CheA were identified.

**Figure 6:**
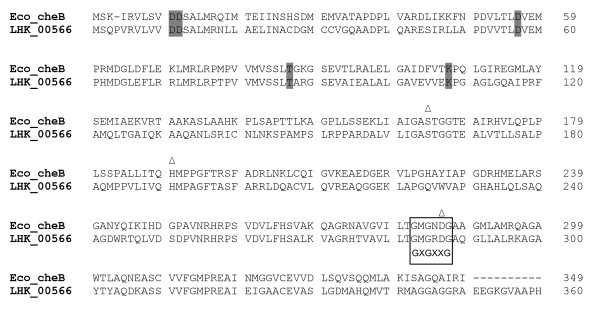
**Amino acid sequence alignment of *L. hongkongensis *and *E. coli *CheBs**. The 5 conserved aspartate, threonine and lysine residues also found in CheY are shaded. The three conserved residues of the catalytic site Ser164, His190 and Asp286 in *E. coli *CheB are marked by triangles above the residues and the GXGXXG nucleotide-binding-fold consensus sequences of other CheB marked in open box.

Other Che proteins are believed to be involved in the regulation of bacterial chemotaxis, although the exact function of some are not fully understood. Among them, CheB is known to work in conjunction with CheR in the reversible methylation of the MCPs. CheR is a constitutively active methyltransferase which methylates the conserved glutamine residues of MCPs, while the methylesterase CheB is responsible for demethylation [49,50]. Similar to CheY, the CheB of *L. hongkongensis *also contained the five conserved amino residues of response regulators. In addition, three conserved residues of the catalytic site, serine at position 164, histidine at position 190 and aspartate at position 286, and the GXGXXG nucleotide-binding-fold sequences conserved among CheB proteins were also present (Figure 6) [51].

Similar multiple copies of chemosensory transducer proteins have also been reported in *C. violaceum *and *Rhodobacter sphaeroides *[46,48]. Interestingly, the organization of the first cluster in *L. hongkongensis*, CA, was similar to one of the three clusters, cluster 3, in *C. violaceum*, although some of the genes were in opposite coding direction. In *R. sphaeroides*, it has been shown that some of the multiple copies of Che proteins are essential (e.g. CheA2) while others are not (e.g. CheA1) although the multiple chemosensory protein homologues are not redundant [46,52]. Further studies are required to investigate the differential function of the multiple copies of chemosensory transducer proteins in *L. hongkongensis*.

#### Flagellar proteins in *L. hongkongensis*

A total of 40 CDSs, arranged in six gene clusters, were likely involved in the biosynthesis of flagella in *L. hongkongensis *(Table 5). These six clusters, FA, FB, FC, FD, FE and FF, encoded 11, 3, 5, 2, 16 and 3 genes respectively. The organization and gene contents of the first five clusters were highly similar to five of the seven clusters of flagellar genes (clusters 1, 2, 4, 5 and 7) previously found in *C. violaceum *[48], which is also a motile bacterium found in multiple ecosystems, including water and soil. On the other hand, the pathogenic *Neisseria *species, *Neisseria gonorrhoeae *and *Neisseria meningitides*, which also belong to the same *Neisseriaceae *family, are non-motile with humans being the only host and reservoir, and do not possess flagellar genes.

**Table 5:** CDSs involved in flagella biosynthesis in *L. hongkongensis *genome

CDS	Gene	Product	Organism with the closest matching sequences	E-value	Identities	Cluster*^a^*
LHK_00436	*flgL*	flagellar hook-associated protein 3	*L. nitroferrum*	1e-59	127/312 (40%)	FA
LHK_00437	*flgK*	flagellar hook-associated protein FlgK	*L. nitroferrum*	1e-109	258/634 (40%)	FA
LHK_00438	*flgJ*	flagellar rod assembly protein/muramidase FlgJ	*L. nitroferrum*	3e-68	144/296 (48%)	FA
LHK_00439	*flgI*	flagellar basal body P-ring protein	*C. violaceum*	1e-95	197/294 (67%)	FA
LHK_00440	*flgH*	flagellar L-ring protein	*L. nitroferrum*	4e-60	122/231 (52%)	FA
LHK_00441	*flgG*	flagellar basal-body rod protein FlgG	*Ralstonia pickettii*	2e-92	162/260 (62%)	FA
LHK_00442	*flgF*	flagellar basal-body rod protein FlgF	*L. nitroferrum*	1e-75	143/246 (58%)	FA
LHK_00443	*flgE*	flagellar basal body FlaE domain-containing protein	*Pseudomonas putida*	4e-76	212/598 (35%)	FA
LHK_00444	*flgD*	flagellar hook capping protein	*L. nitroferrum*	8e-38	88/240 (36%)	FA
LHK_00445	*flgC*	flagellar basal-body rod protein flgC	*C. violaceum*	2e-49	92/136 (67%)	FA
LHK_00446	*flgB*	flagellar basal-body rod protein FlgB	*L. nitroferrum*	2e-41	89/136 (65%)	FA
LHK_00584	*flgN*	FlgN family protein	*C. violaceum*	2e-15	48/131 (36%)	FB
LHK_00585	*flgM*	anti-sigma-28 factor, FlgM	*L. nitroferrum*	4e-09	36/59 (61%)	FB
LHK_00586	*flgA*	flagella basal body P-ring formation protein FlgA	*L. nitroferrum*	2e-36	85/206 (41%)	FB
LHK_00781	*fliA*	RNA polymerase sigma factor for flagellar operon	*C. violaceum*	5e-89	165/242 (68%)	FC
LHK_00782	*fleN*	flagellar synthesis regulator FleN	*L. nitroferrum*	3e-49	121/268 (45%)	FC
LHK_00783	*flhF*	flagellar biosynthesis regulator FlhF	*C. violaceum*	1e-119	250/504 (49%)	FC
LHK_00784	*flhA*	flagellar biosynthesis protein FlhA	*L. nitroferrum*	0	519/682 (76%)	FC
LHK_00785	*flhB*	flagellar biosynthetic protein FlhB	*L. nitroferrum*	2e-136	226/378 (59%)	FC
LHK_02206	*motB*	OmpA/MotB domain protein	*L. nitroferrum*	6e-111	206/273 (75%)	FD
LHK_02207	*motA*	flagellar motor protein MotA	*L. nitroferrum*	9e-123	213/286 (74%)	FD
LHK_02348	*fliR*	flagellar biosynthetic protein FliR	*L. nitroferrum*	1e-60	142/258 (55%)	FE
LHK_02349	*fliQ*	flagellar biosynthetic protein FliQ	*L. nitroferrum*	6e-24	65/89 (73%)	FE
LHK_02350		GCN5-related N-acetyltransferase	*Methylocella silvestris*	5e-09	47/150 (31%)	FE
LHK_02351	*fliP*	flagellar biosynthesis protein FliP	*C. violaceum*	7e-95	178/252 (70%)	FE
LHK_02352	*fliO*	flagellar protein FliO	*C. violaceum*	2e-16	52/100 (52%)	FE
LHK_02353	*fliN*	flagellar motor switch protein FliN	*L. nitroferrum*	2e-54	111/140 (79%)	FE
LHK_02354	*fliM*	flagellar motor switch protein FliM	*L. nitroferrum*	3e-160	272/327 (83%)	FE
LHK_02355	*fliL*	flagellar fliL transmembrane protein	*C. violaceum*	2e-28	64/136 (47%)	FE
LHK_02356	*fliK*	flagellar hook-length control protein	*Nitrosomonas europaea*	5e-18	41/108 (37%)	FE
LHK_02357	*fliJ*	flagellar export protein FliJ	*L. nitroferrum*	3e-20	64/142 (45%)	FE
LHK_02358	*fliI*	flagellar protein export ATPase FliI	*L. nitroferrum*	0	331/453 (73%)	FE
LHK_02359	*fliH*	flagellar assembly protein FliH	*L. nitroferrum*	8e-32	109/275 (39%)	FE
LHK_02360	*fliG*	flagellar motor switch protein FliG	*L. nitroferrum*	2e-148	261/332 (78%)	FE
LHK_02361	*fliF*	flagellar M-ring protein FliF	*L. nitroferrum*	0	339/585 (57%)	FE
LHK_02362	*fliE*	flagellar hook-basal body complex subunit FliE	*L. nitroferrum*	5e-27	69/110 (62%)	FE
LHK_02363		two component, sigma54 specific, transcriptional regulator, Fis family	*L. nitroferrum*	2e-143	279/450 (62%)	FE
LHK_02703	*fliD*	flagellar hook-associated 2 domain protein	*L. nitroferrum*	5e-45	136/445 (30%)	FF
LHK_02704	*flaG*	FlaG flagellar protein	*Janthinobacterium *sp. Marseille	2e-11	38/105 (36%)	FF
LHK_02705	*fliC*	flagellin domain-containing protein	*Acidovorax *sp.	2e-73	159/288 (55%)	FF

*^a^*The flagellar proteins were arranged in six gene clusters, FA, FB, FC, FD, FE and FF (flagellar A, B, C, D, E and F clusters)

A bacterial flagellum is typically composed of three parts, the filament formed by flagellin subunits, basal body attached to the bacterial cell membrane, and the hook which links between the filament and basal body [53]. All the major proteins that form these flagellar components were present in the *L. hongkongensis *genome. They included FliC and FliD which form the major part of the filament; FlgE, FlgK and FlgL which form the hook and hook-filament junction; and Flg B, FlgC, FlgH, FlgI, FlhA, FlhB, FliF, FliG, FliH, FliI, FliM, FliN, FliO, FliP, FliQ, FliR, MotA and MotB which form the basal body and flagellar-motor complex. Putative regulators of these flagellar proteins were also identified. FlgD and FliK are regulators of the hook component FlgE. FlgA, FlgN (both being chaperon proteins) and FliJ are involved in export of flagellar components. The anti-sigma factor gene FlgM and σ28 FliA that regulates late gene products were also present. However, similar to *C. violaceum*, the *L. hongkongensis *genome lacked the FlhDC operon genes, suggesting that the regulation of flagellar protein expression is controlled by FlgM/FliA in this group of bacteria.

#### Quorum sensing in *L. hongkongensis*

In addition to chemotaxis through which bacteria can rapidly adapt to environmental changes, quorum sensing is another way to assess the environment and to recognize the host. Quorum sensing is a signaling system through which bacteria can communicate among themselves by the production of and response to chemical signals called autoinducers [54]. In response to the changing concentrations of these autoinducers, downstream gene expression can be regulated. This cell-to-cell communication system, first identified in *Vibrio harveyi *in the regulation of bioluminescence, is now known to exist in diverse bacteria, especially those that reside in the gastrointestinal tract where recognition of the host may be important for survival and virulence gene expression [54,55]. Among the three major quorum-sensing mechanisms, including the LuxR-I, LuxS/AI-2, and AI-3/epinephrine/norepinephrine systems, known to be utilized by enteric bacteria, only the latter was found in the *L. hongkongensis *genome, suggesting that this system played a major role in quorum-sensing in the bacterium [14].

The AI-3/epinephrine/norepinephrine system is involved in inter-kingdom cross-signaling and regulation of virulence gene transcription and motility [54]. This mechanism is best characterized in enterohemorrhagic *E. coli *(EHEC) which causes fatal hemorrhagic colitis and hemolytic uremic syndrome. It has been shown that the locus of enterocyte effacement (LEE), an important virulence factor in EHEC, and the flagellar genes of EHEC are regulated by the AI-3 system which involves AI-3 produced by the commensal gastrointestinal microflora and/or epinephrine/norepinephrine produced by the host [56,57]. The AI-3 system has also been implicated in biofilm formation in enteropathogenic *E. coli *(EPEC) [58]. Clarke et al. have recently identified the protein, QseC that binds to AI-3 and epinephrine/norepinephrine, suggesting its involvement in the AI-3 system [59]. QseC belongs to a two-component system, QseB/C, in which QseC is the sensor kinase and QseB the response regulator. QseB/C has also been shown to be involved in activation of the flagella regulon and virulence in a rabbit model for EHEC [59,60]. The *L. hongkongensis *genome contained two sets of genes, LHK_00329/LHK_00328 and LHK_1812/LHK_1813, homologous to *qseB/qseC *[14], most closely related to homologues in *C. violaceum *and *Azoarcus *sp. strain BH72 respectively. The two *qseB *genes in *L. hongkongensis *possessed the response regulator receiver domain (PF00072) and the C-terminal domain of transcriptional regulatory protein (PF00486) previously found in the QseB of *E. coli*. The two *qseC *genes in *L. hongkongensis *also contained the His Kinase A (phosphoacceptor) domain (PF00512) and the histidine kinase-, DNA gyrase B-, and HSP90-like ATPase domain (PF02518) previously identified in the QseC of *E. coli*. The presence of two copies of *qseB/qseC *suggested that the AI-3 system may be an important mechanism for adaptation to the changing environment and animal hosts for *L. hongkongensis*.

## Conclusions

A large number of diverse transporters (n = 457), including those from all seven major transporter categories, were identified in the *L. hongkongensis *genome. A diversity of genes involved in chemotaxis, motility and quorum sensing were also found. This suggested that the ability to transport various substances plays an important role in the physiology or survival of *L. hongkongensis*, which may also utilize a complex system to mediate chemotaxis response and adapt to and survive in the rapidly changing environments. In particular, the bacterium is unique among closely related members of *Neisseriaceae *family in possessing higher number of proteins related to transport of ammonium, urea and dicarboxylate, which may reflect the importance of nitrogen and dicarboxylate metabolism in *L. hongkogensis *which is assacharolytic. Structural modeling of two C_4_-dicarboxylate transporters showed that they possessed similar structures to the determined structures of other DctP-TRAP transporters, but one with a rarely seen disulfide bond. A large number of ABC transporters were also identified. These suggest that the bacterium may be able to transport a wide variety of substrates including antibiotics, dyes, detergents, fatty acids, bile salts, organic solvents, ions, amino acids, drugs, heavy metals such as nickel and cobalt, nucleobase, C_4_-dicarboxylates and other metabolites. Diverse mechanisms for iron transport, including hemin transporters for iron acquisition from host proteins, were identified, suggesting that the bacterium may adapt to iron limitation present in human host. Using blastp of all transporters against rcsb pdb, many of these genes were also found to have homolgous proteins of high sequence identities with known structures (data not shown). The large number of chemosensory transducer proteins, many having multiple copies arisen from both horizontal transfer events and gene duplications, may constitute a complex transducer system for mediating chemotaxis response and adapt to environmental changes. The presence of two copies of *qseB/qseC *homologs suggests that *L. hongkongensis *may use the AI-3 system for cross-kingdom quorum-sensing and regulation of potential virulence factors. Further studies are required to better characterize the precise target substance for transport proteins of interest, and the targets regulated by *qseB/qseC *in *L. hongkongensis*, which may shed light on its potential mechanisms for pathogenicity. Structural modeling can be a useful tool to provide useful structural insights about these genes in *L. hongkongensis*.

## Methods

Transport genes were identified and classified according to Transport Classification Database TCDB http://www.tcdb.org/ and manual annotation. These CDSs were from COG C (Energy production and conversion), COG D (Cell cycle control, cell division, chromosome partitioning), COG E (Amino acid transport and metabolism), COG F (Nucleotide transport and metabolism), COG G (Carbohydrate transport and metabolism), COG H (Coenzyme transport and metabolism), COG I (Lipid transport and metabolism), COG J (Translation, ribosomal structure and biogenesis), COG K (Transcription), COG L (Replication, recombination and repair), COG M (Cell wall/membrane/envelope biogenesis), COG N (Cell motility), COG O (post-translational modification, protein turnover, chaperones), COG P (Inorganic ion transport and metabolism), COG Q (Secondary metabolites biosynthesis, transport and catabolism), COG R (General function prediction only), COG S (Function unknown), COG T (Signal transduction mechanisms), COG U (Intracellular trafficking, secretion and vesicular transport) and COG V (Defense mechanisms). CDSs that were classified to COG N (cell motility) and COG T (signal transduction mechanisms), and COG M (cell wall/membrane/envelope biogenesis) were manually annotated for identification of genes related to chemotaxis, motility and quorum sensing. CDSs from other COGs were searched for additional genes using keywords: chemotaxis, che, MCP, flagellar etc. All putative genes were studied by manual curation based on the BLASTx result or multiple alignments. Phylogenetic relationships were determined using Clustal × version 1.81. Protein family analysis was performed using PFAM [61]. Results were also compared to those of *N. gonorrhoeae, N. meningitidis, C. violaceum*, which were the other bacterial species in the *Neisseriaceae *family with complete genome sequences available, where appropriate [29,62-70]. Genes encoding TRAP transporters were located and annotated as described above. Sequence analysis for the presence of signal peptide and transmembrane domains were performed using SignalP v3.0 and TMHMM v2.0 servers respectively [71,72]. Identification of homologs in other bacteria was performed by using BLASTP sequence similarity search against the nr database in NCBI GenBank. The predicted sequences of mature SBPs were submitted to the I-TASSER server for homology modeling using default parameters and available structures of several DctP-type SBP homologs (PDB code: 3B50, 2XA5, 3GYY, 3FXB, 2HPG, and 2CEY) as templates [73]. If multiple homology models were returned, then the best model was selected for further analysis based on the C-score. Quality assessment of the homology model was performed using PROCHECK [74] and ProSA-web [75]. Presence and connectivity of disulfide bonds in the protein were predicted using the DiANNA v1.1 server [76]. Structural alignment of the homology models of SBPs in *L. hongkongensis *and related structures in Protein Data Bank (http://www.pdb.org) was performed using the MatchMaker tool of UCSF Chimera with selected structures (PDB code: 2HZK, 2CEY, 2VPN, 2PFZ, 2PFY, and 2ZZV) [77]. Molecular images were generated using UCSF Chimera.

## List of abbreviations

ABC: ATP-binding cassette; ATP: Adenosine-5'-triphosphate; BFR: Bacterioferritin; CDS(s): Coding sequences(s); COG: Clusters of orthologous group; CPS-E: Capsular polysaccharide export; CrcB: Camphor resistance; DAACS: Dicarboxylate/amino acid:cation (Na or H) Symporter; DASS: Divalent Anion:Na+ Symporter; Dcu: C_4_-dicarboxylate uptake; DNA: Deoxyribonucleic acid; DsbB: Disulfide bond oxidoreductase B; DsbD: Disulfide bond oxidoreductase D; EHEC: Enterohemorrhagic *E. coli*; EPEC: Enteropathogenic *E. coli*; EI: Enzyme I; FAT: Fatty acid transporter; G: Guanine; HAMP: Histidine kinase adenylyl cyclase MCP and phosphatase; HCC: HlyC/CorC; LEE: Locus of enterocyte effacement; MCP(s): Methyl-accepting chemotaxis protein(s); MFP: Membrane fusion protein; MFS: Major facilitator superfamily; PAS: Plasmid achromobacter secretion; PMO: Prokaryotic molybdopterin-cont; P-P-bond: Diphosphate bond; PTS: Phosphotransferase system; RND: Resistance-nodulation-cell-division; TCDB: Transport protein database; TerC: Tellurium ion resistance; TRAP-T: Tripartite ATP-independent periplasmic transporter; VISP: Putative type VI symbiosis/virulence secretory pathway.

## Competing interests

The authors declare that they have no competing interests.

## Authors' contributions

PCYW, KYY and SKPL designed and supervised the study. RYYF, GKMW and JLLT annotated the genome. HT and KHS performed bioinformatics analysis. SKPL, RYYF and GKMW drafted the manuscript. All authors read, corrected and approved the final manuscript.
